# EXPERIENCES OF SENIOR SCHOLARS AND PROFESSORS EMERITI: FOCUS GROUP RESULTS

**DOI:** 10.1093/geroni/igae098.0470

**Published:** 2024-12-31

**Authors:** Michelle Porter, William Kops, Cornelia Kauenhowen

**Affiliations:** University of Manitoba, Winnipeg, Manitoba, Canada; University of Manitoba, Winnipeg, Manitoba, Canada; University of Manitoba, Winnipeg, Manitoba, Canada

## Abstract

In this study we were interested in learning about the experiences of Senior Scholars and Professors Emeriti at the University of Manitoba (UM), as part of a larger age-friendly university project on these term and lifetime positions for retired academics. Focus groups were held online with 11 women and men. The interview guide, developed based on earlier findings from a survey of a larger group of individuals in these same positions, included questions on their reasons for attaining the positions, benefits, challenges, and processes. Six themes were identified from the data collected: 1) commitment, 2) connection/access, 3) variability, 4) overlooked, 5) clarity (lack of) and 6) opportunity. 1) Participants indicated they are committed to their departments and colleagues locally, nationally and internationally. 2) Connections to colleagues and resources are important and appreciated (e.g., email, computer technical support, office and lab space). 3) Despite being at the same university, experiences were not consistent in how they acquired and subsequently renewed positions. 4) While there were many positive experiences shared, some respondents expressed disappointment, concern, anger and even fear, as well as having a sense of not being supported or even ignored. 5) There is an apparent misunderstanding of the designations, access to resources, and resulting expectations and roles. 6) Finally, all were positive about the opportunities to make changes to: nurture a sense of belonging of Senior Scholars and Professors Emeriti; acknowledge their efforts and achievements; create opportunities for increased participation; and ultimately foster a model workplace for retired academics.

